# Dietary acid load, alternative healthy eating index score, and bacterial vaginosis: is there any association? A case-control study

**DOI:** 10.1186/s12879-022-07788-3

**Published:** 2022-10-27

**Authors:** Morvarid Noormohammadi, Ghazaleh Eslamian, Seyyedeh Neda Kazemi, Bahram Rashidkhani

**Affiliations:** 1 Department of Nutrition, School of Public Health, Iran University of Medical Sciences, Tehran, Iran; 2 Student Research Committee, Faculty of Public Health Branch, Iran University of Medical Sciences, Tehran, Iran; 3 Department of Cellular and Molecular Nutrition, Faculty of Nutrition and Food Technology, National Nutrition and Food Technology Research Institute, Shahid Beheshti University of Medical Sciences, No 7, Hafezi St., Farahzadi Blvd, P.O.Box: 19395-4741, 1981619573 Tehran, Iran; 4 Department of Obstetrics and Gynecology, School of Medicine, Preventative Gynecology Research Center, Imam Hossein Hospital, Shahid Beheshti University of Medical Sciences, Tehran, Iran; 5 Department of Community Nutrition, Faculty of Nutrition and Food Technology, National Nutrition and Food Technology Research Institute, Shahid Beheshti University of Medical Sciences, Tehran, Iran

**Keywords:** Bacterial vaginosis, Dietary patterns, Alternative healthy eating index, Dietary acid load, Plant-based diet

## Abstract

**Background:**

Changing the dietary pattern may be an alternative treatment for bacterial vaginosis, the prevalent vaginal infection in women.

**Methods:**

One hundred and forty-three bacterial vaginosis-affected women diagnosed by Amsel criteria and 151 healthy controls aged 18 to 45 entered the current case-control research. To calculate the alternative healthy eating index and dietary acid load score, food consumption was recorded with an accurate and precise food frequency questionnaire. The dietary acid load was measured by potential renal acid load (PRAL) and net endogenous acid production (NEAP) indices. Using logistic regression models, the association between the alternative healthy eating index and dietary acid load score with bacterial vaginosis was investigated.

**Results:**

The last tertile of the alternative healthy eating index had a 75% decreased odds of experiencing bacterial vaginosis in the adjusted model (adjusted odds ratio (aOR) = 0.25, 95% confidence interval (CI) = 0.12–0.53, P for trend = 0.001). Besides, vegetables (aOR = 0.34, 95% CI = 0.17–0.69, P for trend = 0.003), nuts and legumes (aOR = 0.44, 95% CI = 0.23–0.87, P for trend = 0.028), and meats (aOR = 0.31, 95% CI = 0.16–0.60, P for trend = 0.001) intake was linked to a decreased bacterial vaginosis odds. However, sugar-sweetened beverages and fruit juice (aOR = 3.47, 95% CI = 1.68–7.17, P for trend < 0.001), trans fatty acids (aOR = 2.29, 95% CI = 1.18–4.43, P for trend = 0.005), and sodium (aOR = 3.44, 95% CI = 1.67–7.06, P for trend = 0.002) intake were directly associated with bacterial vaginosis odds. There was no evidence of a link between dietary acid load and bacterial vaginosis.

**Conclusion:**

According to the present study’s findings, there is no correlation between dietary acid load and the likelihood of developing bacterial vaginosis. However, following a plant-based dietary pattern based on the healthy eating index may lead to a lower odds of bacterial vaginosis.

## Background

Bacterial vaginosis (BV) is one of the vaginal infections in women of reproductive age with a high prevalence. It is defined by a shift in the predominance of *Lactobacillus* in the vaginal microbiota to anaerobic species such as *Atopobium*, *Gardnerella*, *Prevotella*, and *Papillibacter* [1]. BV has been considered a microbiological and immunological issue [2]. Patients with BV often experience vaginal discharge, itching, and burning without redness [3]. Although 50% of affected individuals exhibit no symptoms, BV associates with obstetric and gynecologic problems, such as pelvic inflammatory disease and endometriosis [4, 5], affecting the host immune system [6]. The etiology of BV is unknown; even so, risk factors are ethnic origin (black/Hispanic), frequent vaginal douching, smoking, and numerous sex partners without condom usage [7]. Oral contraceptives, such as oral estrogen, decrease BV rates [7]. Half of the women will experience recurrence after medical treatment in 12 months, and recurrent BV treatment is challenging [7]. Therefore, an individual’s diet may serve as an alternative treatment objective [8]. In this regard, in previous studies, researchers have stated a positive link between processed foods consumption, high glycemic index (GI), and high glycemic load (GL) foods, and an inverse association between a rich dietary fiber pattern and some components of the Mediterranean diet with the odds of BV [9–11].

The Healthy Eating Index, HEI, is a tool for determining the dietary quality and how well it adheres to the Dietary Guidelines for Americans (DGA) [12]. It has been applied to find and evaluate relationships between diet quality and particular illnesses, including inflammatory disorders [13]. Thoma et al. demonstrated that BV was not linked with HEI values less than 70; nevertheless, there was a connection between HEI values more than 70 and a decrease in BV. However, this finding was restricted in its ability to generalize to other groups due to the sample’s majority being African American and the absence of confounding factors such as physical activity [14]. As a result, it seems that the association between BV and HEI should be reconsidered.

Moreover, the primary difference between vaginal eubiosis (defined by beneficial *lactobacillus*-dominated microbiota) and dysbiosis (defined by an overgrowth of anaerobes species) is that the decrease in lactic acid production leads to increased vaginal pH [15]. Dietary acid or base precursors have been recognized to affect acid-base and pH balance. Protein-rich meals enhance acidity, whereas fruits and vegetables promote alkalinity. A food’s potential renal acid load is its acid- or base-producing capability [16]. Due to the critical role of vaginal pH alteration in the pathogenesis of BV [15], the present study postulated that dietary acid load might have a principal effect on BV progression through vaginal pH. Furthermore, to our knowledge, there has been no investigation into whether dietary acid load and BV are linked. This research aimed to determine the link between the healthy eating index and dietary acid load with BV.

## Methods

### Study population

In the current case-control research, referred participants from the gynecology clinic (Imam Hossein Hospital, Tehran) were chosen, using a convenience sample approach, and provided with written permission to participate. Participants entered the study if they were aged 18 to 45 years and were not pregnant or in menopause. Sample size calculation and other inclusion criteria have been stated in the previous studies [9–11]. Participants who did not answer at least 60% of the food frequency questionnaire (FFQ) items were eliminated, as well as those who reported energy consumption outside of the ± 3 standard deviation (SD) of the mean intake and decided to quit the study.

### Medical assessments

A gynecologist conducted the patient evaluation in order to determine if they had BV or not. The same diagnostic test was done for all participants. As a clinical diagnosis, at least three instances have to meet the Amsel criteria: a homogenous and diluted vaginal discharge, a vaginal pH higher than 4.5, the presence of 20% clue cells when using saline microscopy, and the smell of fish after adding 10% potassium hydroxide to the discharge slide [8, 17–19].

### Data on dietary intake

Food frequency and demographic information forms (age, sex, education level, employment) and health status assessments were completed during the interview with the participants. This FFQ has 168 dietary items, each with a typical portion size and following the Willet method [20]. The validity and reliability of this FFQ have been confirmed [21], and previous studies have utilized this questionnaire to identify dietary patterns [22, 23]. Further details are provided in previous studies [9–11].

### Anthropometric assessments

Participants’ weights were measured in light clothes with a 100-gram accuracy. Then heights were measured when the individual stood in a straight stance, with 1 mm precision. Details are provided in previous studies [9–11]. Body mass index (BMI) was determined by dividing weight (kg) by height squared (m2) (square meters).

All participants were asked to fill out questionnaires and have their anthropometric data assessed by a trained interviewer. To prevent selection bias, the individual who conducted the BV diagnosis was not informed of the patients’ food consumption. To prevent information bias, the responses were collected by a professional surveyor who was blind to the sample findings at the time of the interview. A hospital lab technician also conducted the diagnosis.

### Alternative healthy eating index and dietary acid load

The Alternative Healthy Eating Index, AHEI, is a revised version of the original healthy eating index developed by Kennedy et al. [24]. This approach examined 11 elements: whole grains, fruits, nuts and legumes, vegetables, long-chain n-3 fatty acids (DHA and EPA), polyunsaturated fatty acids (PUFA), sugar-sweetened beverages and fruit juice, red and processed meats, wine, and trans-fat and sodium. Due to a paucity of data on wine and long-chain n-3 fatty acids (DHA and EPA) consumption in our database, we calculated AHEI using nine components in this research. Individuals in the highest decile of whole grains, vegetables, fruits, nuts and legumes, and PUFA got a score of 10, while those who consumed the least received a score of 1. Individuals in the other deciles got equivalent scores.

In comparison, participants who consumed the most sugar-sweetened beverages and fruit juice, trans fatty acids, red and processed meats, and sodium got a score of 1, while those who consumed the least of these components obtained 10. The participant’s total AHEI score was then calculated by adding the results for these ten components. It ranged from 9 to 90.

There are two validated and common measurements for estimation of the potential acid load produced by the consumption of a single food or overall diet and potential renal acid load (PRAL). This is based on dietary protein, phosphorus, potassium, calcium, and magnesium intake [25] and net endogenous acid production (NEAP), which is related to protein and potassium intake [26].:

1) PRAL (mEq/day) = (protein (g/d) × 0.49) + (Phosphorous (mg/d) × 0.037) – (potassium (mg/d) × 0.021) – (Calcium (mg/d) × 0.013) – (magnesium (mg/d) × 0.026).

2) NEAP (mEq/day) = (54.5 × protein (g/d)/potassium (mEq/d))-10.2.

The validity of both PRAL and NEAP scores compared to a 24-h urinary acid load is confirmed [25, 26]. Low and negative PRAL indicate an alkaline diet, opposite to high and positive PRAL, which shows an acidic diet. Similarly, higher NEAP scores indicate an acidic diet [25, 26].

### Statistical analysis

SPSS software version 22 was used to conduct the statistical analysis. For all hypothesis tests, a P-value of 0.05 or less indicated statistical significance. The continuous variables were tested for normal distribution using Q-Q plots and Kolmogorov-Smirnov tests. Frequency and percentages were used to express the categorical characteristics, and the median (interquartile range, IQR) was used for quantitative characteristics. Quantitative data in BV-affected patients and healthy groups were compared using the Student t-test, and the Mann-Whitney test was run for non-normally distributed variables. To compare qualitative features between the case and control groups, the Chi-Square test was used. Quantitative and qualitative confounding factors across AHEI tertiles were evaluated using the Kruskal-Wallis test or χ2 test/Fisher’s exact test, respectively. To examine the relationship between AHEI, DAL, and BV, logistic regression was performed to calculate base and adjusted odds ratios (OR) with 95% CI. All food items were energy-adjusted using the residual method. Besides, age (years), BMI (Kg/m2), waist circumferences, WC (cm), smoking (per day), number of pregnancies, Iron supplementation (yes/no), and physical activity (MET/h/d) were controlled for adjusted ORs.

### Ethics

This research was authorized by the National Nutrition and Food Technology Research Institute, Shahid Beheshti University of Medical Sciences, Tehran, Iran (project NO 99/25,431). The ethics committee code was IR.SBMU.NNFTRI.REC.1399.054. In order to participate in the research, each participant had to sign a written informed consent form. All procedures were conducted according to the latest version of the Helsinki Declaration.

## Results

From 148 BV-affected women and 153 healthy controls, four patients of the case group and two control group participants were excluded because the energy intake was > + 3SD or < -3SD from the mean. One BV-affected woman was excluded from the statistical analysis due to a lack of data. Therefore, 143 BV-affected women and 151 healthy controls were included in the study. Participation rates were 96.6% among cases and 98.7% among controls. The median (Q1-Q3) age in the case and control group participants was 30 (25–33) and 32 (24–37) years, respectively. General characteristics of the case and control group participants according to the AHEI score tertiles are presented in Table 1. There was a significant difference in the age of control (P-value = 0.020) and case (P-value < 0.001) group participants across the AHEI tertiles. Besides in the case group, participant were significantly different according to smoking (P-value = 0.003), number of pregnancies (P-value = 0.049), waist circumference (P-value = 0.022), and iron supplement consumption (P-value = 0.014) (Table 1).

**Table 1:** General characteristics separately across tertiles of AHEI-2010

Characteristic^a^	CONTROL				CASE			
	Tertile 1 N = 53	Tertile 2 N = 51	Tertile 3 N = 47	P Value^b^	Tertile 1 N = 83	Tertile 2 N = 46	Tertile 3 N = 14	P Value^b^
Age, year, median (Q_1_-Q_3_)	26 (24–33)	35 (24–40)	33 (25–36)	0.020*	29 (25–33)	30 (24–33)	37 (30–39)	< 0.001*
Familial history of BV, Yes	13 (24.5)	13 (25.5)	11 (23.4)	0.972	47 (56.6)	23 (50)	7 (50)	0.735
Education				0.546				0.136
Primary/secondary school	13 (24.5)	10 (19.6)	16 (34)		17 (20.5)	14 (30.4)	5 (35.7)	
Undergraduate	24 (45.3)	27 (52.9)	19 (40.4)		42 (50.6)	26 (56.5)	8 (57.1)	
Graduate	16 (30.2)	14 (27.5)	12 (25.5)		24 (28.9)	6 (13)	1 (7.2)	
Cigarette per day				0.761				0.003*
0	52 (98.1)	51 (100)	46 (97.9)		60 (72.3)	44 (95.7)	14 (100)	
1–2	0	0	0		10 (12)	2 (4.3)	0	
≥ 3	1 (1.9)	0	1 (2.1)		13 (15.7)	0	0	
Employment status, Employed	14 (26.4)	11 (21.6)	17 (36.2)	0.262	26 (31.3)	14 (30.4)	3 (21.4)	0.827
Monthly family income, < 250 US $	43 (81.1)	41 (80.4)	37 (78.7)	0.954	66 (79.5)	35 (76.1)	9 (64.3)	0.440
Number of pregnancies				0.208				0.049*
0	30 (56.6)	21 (41.2)	19 (40.4)		41 (49.4)	20 (43.5)	2 (14.3)	
1–2	18 (34)	22 (43.1)	25 (53.2)		35 (42.2)	23 (50)	8 (57.1)	
≥ 3	5 (9.4)	8 (15.7)	3 (6.4)		7 (8.4)	3 (6.5)	4 (28.6)	
Menstrual cycle, Regular	30 (56.6)	40 (78.4)	32 (68.1)	0.059	55 (66.3)	28 (60.9)	11 (78.6)	0.506
Number of sexual partners in the previous month				0.053				0.127
0	23 (43.4)	19 (37.3)	11 (23.4)		31 (37.4)	16 (34.8)	1 (7.1)	
1	27 (50.9)	31 (60.8)	36 (76.6)		49 (59)	27 (58.7)	13 (92.9)	
≥ 2	3 (5.7)	1 (2)	0		3 (3.6)	3 (6.5)	0	
Physical activity (MET/h/d), median (Q_1_-Q_3_)	40.5 (36–46)	39.7 (35.7–46.7)	40.6 (37.3–46.9)	0.891	39.8 (35.1–44.3)	39.8 (36.2–44.8)	41.2 (35.9–49.9)	0.770
Weight (Kg)	64 (56-71.5)	65 (58–72)	67 (57–76)	0.631	69 (60–79)	66 (64–75)	69 (59.75–76.25)	0.710
Height (cm)	162 (158.5–165)	161 (157–165)	162 (158–165)	0.424	163 (159–167)	164 (160–167)	162.5 (157-169.75)	0.662
BMI (Kg/m^2^), median (Q1-Q3)	23.9 (21.8–26.4)	24.8 (22.7–28.0)	24.6 (22-28.6)	0.326	26.6 (22.9–29.1)	25.1 (23.7–28.3)	25.4 (24.0-28.5)	0.283
WC (cm), median (Q_1_-Q_3_)	78 (68.5–90)	82 (77–95)	84 (71–95)	0.402	87 (76–95)	85 (67.7–90)	100 (92-103.5)	0.022*
Supplements								
Calcium 500 mg/day, Yes	10 (18.9)	8 (15.7)	6 (12.8)	0.706	6 (7.2)	2 (4.3)	2 (14.3)	0.367
Folate 400 µg/day, Yes	8 (15.1)	7 (13.7)	7 (14.9)	0.978	17 (20.5)	11 (23.9)	2 (14.3)	0.787
Vitamin D 50,000 IU/month, Yes	23 (43.4)	18 (35.3)	25 (53.2)	0.203	24 (28.9)	22 (47.8)	3 (21.4)	0.058
Iron 30 mg/day, Yes	11 (20.8)	17 (33.3)	13 (27.7)	0.352	12 (14.5)	14 (30.4)	0	0.014*
Omega 3 1–3 gr/day, Yes	3 (5.7)	2 (3.9)	3 (6.4)	0.905	3 (3.6)	4 (8.7)	0	0.329

a Values are No (%)unless otherwise noted

b Using the Kruskal-Wallis test or χ2 test/Fisher’s exact test, as appropriate.

AHEI-2010, Alternative Healthy Eating Index-2010; BV, Bacterial Vaginosis; BMI, Body Mass Index; WC, Waist circumference

Investigating the association between AHEI and BV showed that in Model 1, the odds of BV was 81% lower in the last tertile of AHEI (which was equal to more than 58) (OR = 0.19, 95%CI = 0.10–0.39, P for trend < 0.001). This significantly inverse link remained in the adjusted model (aOR = 0.25, 95%CI = 0.12–0.53, P for trend = 0.001). To consider healthy eating index components, odds of BV was 72% and 66% lower in the last tertile of vegetables group (equals to more than 415 gr/day) in both base and adjusted models (OR = 0.28, 95%CI = 0.15–0.54, P for trend < 0.001, and aOR = 0.34, 95%CI = 0.17–0.69, P for trend = 0.003, respectively). High consumption of nuts and legumes group (equals to more than 77 gr/day) was associated with 64% and 56% lower odds of BV in base and adjusted models (OR = 0.36, 95%CI = 0.19–0.67, P for trend = 0.002, and aOR = 0.44, 95%CI = 0.23–0.87, P for trend = 0.028, respectively). In Model 1, a significant association was observed between odds of BV and high consumption of sugar-sweetened beverages and fruit juice group (equals to more than 50 gr/day) and trans fatty acids group (equal to more than 1.8 gr/day). In the adjusted model, BV odds was more than 3 times higher in the last tertile of sugar-sweetened beverages and fruit juice group (aOR = 3.47, 95%CI = 1.68–7.17, P for trend < 0.001), and more than 2 times higher in the last tertile of trans fatty acids group (aOR = 2.29, 95%CI = 1.18–4.43, P for trend = 0.005). Participants in the second tertile of the sodium group (equal to more than 2900 mg/day) and those in the last tertile (equal to more than 3800 mg/day) had about 3 times higher odds of BV in both base (P for trend < 0.001) and adjusted models (P for trend = 0.002). Interestingly, high consumption of red and processed meats (equals to more than 100 gr/day) was associated with lower odds of BV (aOR = 0.31, 95%CI = 0.16–0.60, P for trend = 0.001) (Table 2).

**Table 2:** Adjusted odds ratio (OR) estimates and 95% confidence intervals (CIs) for bacterial vaginosis depending on the tertile of AHEI-2010*

AHEI and its parameters	Tertiles of AHEI			*P* for Trend*
	1st	2nd	3rd	
**AHEI-2010 score (energy-adjusted)**				
Number of BV patients/Number of healthy participants	83 / 53	46 / 51	14 / 47	
Model 1 ^†^	1.00 (Ref.)	0.62 (0.36–1.06)	0.19 (0.10–0.39)	< 0.001
Model 2 ^‡^	1.00 (Ref.)	0.86 (0.48–1.53)	0.25 (0.12–0.53)	0.001
**Fruits group (energy-adjusted, grams per day)**				
Number of BV patients/Number of healthy participants	63 / 50	50 / 51	30 / 50	
Model 1 ^†^	1.00 (Ref.)	0.90 (0.49–1.65)	0.51 (0.28–0.93)	0.028
Model 2 ^‡^	1.00 (Ref.)	1.17 (0.60–2.28)	0.63 (0.33–1.22)	0.156
**Vegetable group (energy-adjusted, grams per day)**				
Number of BV patients/Number of healthy participants	73 / 50	50 / 51	20 / 50	
Model 1 ^†^	1.00 (Ref.)	0.69 (0.41–1.19)	0.28 (0.15–0.54)	< 0.001
Model 2 ^‡^	1.00 (Ref.)	0.71 (0.40–1.28)	0.34 (0.17–0.69)	0.003
**Whole grains group (energy-adjusted, grams per day)**				
Number of BV patients/Number of healthy participants	54 / 50	59 / 51	30 / 50	
Model 1 ^†^	1.00 (Ref.)	1.22 (0.68–2.19)	0.63 (0.34–1.17)	0.161
Model 2 ^‡^	1.00 (Ref.)	1.23 (0.65–2.35)	0.60 (0.30–1.20)	0.156
**Nuts and legumes group (energy-adjusted, grams per day)**				
Number of BV patients/Number of healthy participants	68 / 50	52 / 51	23 / 50	
Model 1 ^†^	1.00 (Ref.)	0.98 (0.53–1.81)	0.36 (0.19–0.67)	0.002
Model 2 ^‡^	1.00 (Ref.)	1.20 (0.62–2.31)	0.44 (0.23–0.87)	0.028
**PUFA (energy-adjusted, grams per day)**				
Number of BV patients/Number of healthy participants	39 / 50	50 / 51	54 / 50	
Model 1 ^†^	1.00 (Ref.)	1.38 (0.76–2.49)	1.41 (0.79–2.52)	0.253
Model 2 ^‡^	1.00 (Ref.)	1.67 (0.87–3.20)	1.79 (0.94–3.41)	0.085
**Red and processed meats group (energy-adjusted, grams per day)**				
Number of BV patients/Number of healthy participants	77 / 50	45 / 51	21 / 50	
Model 1 ^†^	1.00 (Ref.)	0.61 (0.35–1.05)	0.26 (0.14–0.49)	< 0.001
Model 2 ^‡^	1.00 (Ref.)	0.60 (0.33–1.08)	0.31 (0.16–0.60)	0.001
**Sugar-sweetened beverages and fruit juice group (energy-adjusted, grams per day)**				
Number of BV patients/Number of healthy participants	28 / 50	34 / 51	81 / 50	
Model 1 ^†^	1.00 (Ref.)	1.59 (0.77–3.27)	3.55 (1.83–6.87)	< 0.001
Model 2 ^‡^	1.00 (Ref.)	1.55 (0.71–3.37)	3.47 (1.68–7.17)	< 0.001
**Trans Fatty Acids group (energy-adjusted, milligrams per day)**				
Number of BV patients/Number of healthy participants	32 / 50	25 / 51	86 / 50	
Model 1 ^†^	1.00 (Ref.)	0.90 (0.44–1.82)	3.02 (1.65–5.55)	< 0.001
Model 2 ^‡^	1.00 (Ref.)	0.80 (0.38–1.68)	2.29 (1.18–4.43)	0.005
**Sodium group (energy-adjusted, milligrams per day)**				
Number of BV patients/Number of healthy participants	17 / 50	62 / 51	64 / 50	
Model 1 ^†^	1.00 (Ref.)	4.32 (2.15–8.66)	4.10 (2.09–8.07)	< 0.001
Model 2 ^‡^	1.00 (Ref.)	3.36 (1.63–6.95)	3.44 (1.67–7.06)	0.002

^*^ logistic regression model

^†^Base model, adjustment for age (years) and total calories intake (Kcal/d)

^‡^ Additionally adjusted for body mass index (Kg/m^2^), waist circumferences (cm), iron supplementation (yes/no), folate supplementation (yes/no), vitamin D supplementation (yes/no), calcium supplementation (yes/no), physical activity (MET/h/d), number of sexual partners in the previous month, and number of pregnancies, salary (< 250 $/month or 250$/month=<), education (Primary/secondary school, Undergraduate, Graduate), cigarette per day AHEI-2010, Alternative Healthy Eating Index-2010; BV, bacterial vaginosis

The association between PRAL and NEAP and the odds of BV, was not statistically significant (Table 3).

**Table 3:** Adjusted odds ratio (OR) estimates and 95% confidence intervals (CIs) for bacterial vaginosis depending on the tertile of dietary acid load*

dietary acid load	Tertiles of dietary acid load			*P* for Trend*
	1st	2nd	3rd	
**PRAL (energy-adjusted, mEq/day)**				
Number of BV patients/Number of healthy participants	46 / 50	40 / 51	57 / 50	
Model 1 ^†^	1.00 (Ref.)	0.87 (0.48–1.57)	1.18 (0.67–2.07)	0.567
Model 2 ^‡^	1.00 (Ref.)	0.81 (0.42–1.55)	1.13 (0.60–2.13)	0.697
**NEAP (energy-adjusted, mEq/day)**				
Number of BV patients/Number of healthy participants	56 / 50	29 / 51	58 / 50	
Model 1 ^†^	1.00 (Ref.)	0.51 (0.28–0.93)	1.01 (0.58–1.75)	0.947
Model 2 ^‡^	1.00 (Ref.)	0.45 (0.23–0.87)	0.94 (0.51-1. 76)	0.742

^*^ logistic regression model

^†^Base model, adjustment for age (years) and total calories intake (Kcal/d)

^‡^Additionally adjusted for body mass index (Kg/m^2^), waist circumferences (cm), iron supplementation (yes/no), folate supplementation (yes/no), vitamin D supplementation (yes/no), calcium supplementation (yes/no), physical activity (MET/h/d), number of sexual partners in the previous month, and number of pregnancies, salary (< 250 $/month or 250$/month=<), education (Primary/secondary school, Undergraduate, Graduate), cigarette per day

PRAL, potential renal acid load; NEAP, net endogenous acid production; BV, bacterial vaginosis

## Discussion

In this current study, the link between dietary acid load and BV was investigated for the first time based on the literature review. No association between dietary acid load and BV was observed. However, adherence to AHEI decreased the odds of BV significantly in base and adjusted models. Similarly, Thoma et al. observed a significant negative association between HEI and BV but only in the unadjusted model [14].

This study showed that in Model 1, the odds of BV in the last tertile of the vegetables, nuts and legumes, and fruits group were lower than the reference tertile. In addition, in the adjusted model, the odds of BV remained inversely and significantly associated with the consumption of vegetables, and nuts and legumes. Noormohammadi et al. showed that two dietary components in the Mediterranean diet, including vegetables and legumes, were associated with lower odds of BV [9]. We have previously shown a negative link between dietary fiber and BV odds [10]. Similarly, in one study by Shivakoti et al. the connection between dietary macronutrient intake and molecular-BV, fiber (rich in fruits, vegetables, and legumes) was negatively associated with the risk of BV [27]. Fiber is a nutrient with prebiotic effects which improves vaginal microbiota by enhancing the *Lactobacillus* species [28]. As functional foods, vegetables, fruits, nuts, and legumes are rich in flavonoids with anti-inflammatory effects resulting in the inhibition of nuclear factor kappa-light-chain-enhancer of activated B cells (NF-κB) [29]. NF-κB is the primary factor in the pro-inflammatory signaling pathways, and its stimulation in different cells in vaginal secretions of those with BV has been shown [30]. Together, these prebiotics have a beneficial effect on intestinal health [31]. Fiber intake may enhance gut barrier integrity, bacterial diversity, and inflammatory responses [32–35], and the vaginal microbiota may be impacted by colonization from the rectum in the vagina [36]. Evidence suggests the effect of functional foods, probiotics, and prebiotics improve vaginal inflammation [14, 37]. Immune tolerance is the condition in which the immunological system is active, tightly controlled, and resistant to self-antigens or to a specific antigen that might cause an immune response in the body [38]. The lack of inflammation in BV may be explained by immune tolerance to healthy and unhealthy vaginal microbiota, primarily obtained from gut microbiota as a consequence of coevolution with humans. This hypothesis is supported by short-chain fatty acids, abundantly generated by intestinal microflora through fermenting dietary fiber, and are known to modulate immune responses [2]. So like the probiotics, prebiotic fiber alters the gut microbiota and may execute its role in lowering inflammation by inducing short-chain fatty acids and bile acids generation and NF-κB signaling pathway suppression [39].

In another study by Neggers et al., severe BV was negatively correlated with folate (vitamin B9) and vitamin E intake [40], vitamins that are found mainly in vegetables and fruits [41, 42]. Vitamin E has antioxidant effects [43–45], and vitamins B9 and E may decrease severe BV risk by improving immunological function [40]. In addition, women lacking micronutrients like beta-carotene and the vitamins D, C, A, and E are more likely to get BV. This deficiency may show low fruit and vegetable consumption. Consuming fresh fruit and vegetables on a regular basis leads to a lower risk of vaginitis [40, 46, 47], and educating patients about vegetable and fruit intake may have a significant effect [47]. In contrast to our study, Thoma et al. did not show any association between HEI and BV odds after adjusting for confounders. However, similar to the Noormohammadi et al. study [10], Thoma et al. showed a direct association between GL and the persistence and acquisition of BV. High-GL foods damage the host’s immune response leading to increased oxidative stress [14]. Healthy elements found in fruits and vegetables, such as fiber, may lessen the adverse effects of high GL diets [48].

This study observed a link between the meat group and reduced odds of BV in both models. According to Verstraelen et al., subclinical iron insufficiency is related to vaginosis-like microbiota during early pregnancy [49]. Iron is a nutrient found in animal meat [50]. Innate and adaptive immunological responses in the vaginal mucosa may cause failure due to iron deficiency. This leads to more susceptibility to infectious diseases [51, 52]. Neggers et al. observed a significant inverse association between protein intake and severe BV [40], a primary macronutrient found in meat [53]. Inversely, processed meats and fast foods are linked with increased BV odds, according to Noormohammadi et al. study [11, 54], which may be due to the rich fat content in these foods and the link between total fat intake and elevated BV [40].

As shown in the results, the odds of BV in the last tertile of the trans fatty acids group were more than doubled in contrast to the reference. We have also shown the direct association between unhealthy dietary patterns, ultra-processed oil, processed foods, and fast foods high in trans fatty acids with BV odds [11, 54]. Neggers et al. declared a significant link between total fat consumption and BV and an association between total fat and saturated fat with severe BV. Consuming fat in high amounts may lead to elevated vaginal pH, which may increase BV risk [40]. High fat intake affects the immunological functions of the gut, and due to the vital role of gut-associated lymphoid tissue on the mucosal immune system, the risk of BV increases [40, 55–58].

According to the results, being in the last tertile of sugar-sweetened beverages and fruit juice, foods with a high GL, would triple BV odds. According to previous studies, BV odds is directly associated with ultra-processed sweets consumption [11], dietary GI, and dietary GL [10]. Similarly, Thoma et al. state that GL directly relates to BV [14]. Foods with a high GL or high GI may fail the immune response toward bacterial colonization via oxidative stress [36, 59].

The vaginal pH alteration is the principal factor in BV pathogenesis [15]; therefore, our study assumed that dietary acid load affects BV progression through vaginal pH. The present study used PRAL and NEAP for dietary acid load calculation. These are two validated and common measurements for dietary acid load estimation [25, 26] that are frequently used in previous epidemiologic studies [60–64]. PRAL is based on dietary protein, phosphorus, potassium, calcium, and magnesium intake [25].NEAP is based on protein and potassium intake [26]. However, no significant association was found between dietary acid load and BV. The association between protein, phosphorus, potassium, calcium, and magnesium intake and BV odds is different from the PRAL and NEAP calculation using these components [25, 26]. Considering the association between micro/macronutrients and the odds of BV would be exciting research suggested for future studies.

This research provides several advantages in a variety of ways. Individuals who provided inaccurate or excessive energy intake were excluded. Included were patients with recently developed BV. An incident case selection strategy would support a causal interpretation and reduce memory bias [65, 66]. High involvement rates were seen in both the cases and controls. Regression models allowed for the modification of many potential variables. A single person performed a diagnostic test in the hospital lab to remove information bias in all groups. A qualified dietitian who was not aware of the diagnosis outcomes also collected the questions.

The current study, however, contains a number of significant flaws. Even if the researchers took every precaution to prevent bias, a case-control design might still provide inaccurate findings due to selection bias, measurement bias, and recollection bias. The many bacteria that cause BV were not looked at in the current study, which is highly recommended for future studies to be considered. The gold standard for diagnosing BV, the Nugent score, was not applied and recommended for future studies [18]. Besides, it is suggested for future research to assess serum and urinary pH. Due to Iran’s cultural and religious restrictions, no statistics on alcohol or opium were collected.

## Conclusion

Dietary acid load is not associated with the odds of BV. However, following a dietary pattern based on the healthy eating index is associated with lower odds of BV. High consumption of vegetables, nuts, legumes, and meats is associated with reduced odds of BV. In addition, there is a direct association between sugar-sweetened beverages and fruit juice, trans fatty acids, and sodium intake with BV odds. Following a plant-based dietary pattern rich in plant-based protein foods, which are also high in fiber, is recommended for women at high risk of BV.

## Data Availability

The corresponding author will provide the data supporting this research’s findings upon a reasonable request.

## Abbreviations

AHEI: Alternative Healthy Eating Index
BMI: Body mass index
BV: Bacterial Vaginosis
DAL: Dietary Acid Load
DGA: Dietary Guidelines for Americans
FFQ: Food frequency questionnaire
GI: Glycemic Index
GL: Glycemic Load
HEI: The Healthy Eating Index
NF-κB: nuclear factor kappa-light-chain-enhancer of activated B cells
PUFA: Polyunsaturated Fatty Acids
WC: Waist circumference

## References

[1] Xiao B, Niu X, Han N, Wang B, Du P, Na R (2016). Predictive value of the composition of the vaginal microbiota in bacterial vaginosis, a dynamic study to identify recurrence-related flora. Sci Rep.

[2] Danielsson D, Teigen PK, Moi H (2011). The genital econiche: focus on microbiota and bacterial vaginosis. Ann N Y Acad Sci.

[3] Chang HH, Larson J, Blencowe H, Spong CY, Howson CP, Cairns-Smith S (2013). Preventing preterm births: analysis of trends and potential reductions with interventions in 39 countries with very high human development index. Lancet.

[4] Reiter S, Kellogg Spadt S (2019). Bacterial vaginosis: a primer for clinicians. Postgrad Med.

[5] Tokyol Ç, Aktepe OC, Cevrioğlu AS, Altındiş M, Dilek FH (2004). Bacterial vaginosis: comparison of Pap smear and microbiological test results. Mod Pathol.

[6] Delbandi A-A, Mahmoudi M, Shervin A, Moradi Z, Arablou T, Zarnani A-H (2020). Higher frequency of circulating, but not tissue regulatory T cells in patients with endometriosis. J Reprod Immunol.

[7] Bagnall P, Rizzolo D (2017). Bacterial vaginosis: A practical review. Jaapa.

[8] Tuddenham S, Ghanem KG, Caulfield LE, Rovner AJ, Robinson C, Shivakoti R, et al. Associations between dietary micronutrient intake and molecular-Bacterial Vaginosis. Reproductive Health. 2019;16(1). 10.1186/s12978-019-0814-6 PMC6806504 31640725

[9] Noormohammadi M, Eslamian G, Kazemi SN, Rashidkhani B (2022). Is there any association between adherence to the Mediterranean Diet and Dietary Total Antioxidant Capacity with Bacterial Vaginosis? Results from a Case–Control study. BMC Womens Health.

[10] Noormohammadi M, Eslamian G, Kazemi SN, Rashidkhani B, Malek S. Association of Dietary Glycemic Index, Glycemic Load, Insulin Index, and Insulin Load with Bacterial Vaginosis in Iranian Women: A Case-Control Study. Infectious Diseases in Obstetrics and Gynecology. 2022;2022. 10.1155/2022/1225544 PMC8970957 35370395

[11] Noormohammadi M, Eslamian G, Kazemi SN, Rashidkhani B, Omidifar F (2022). Association between consumption of ultra-processed foods and bacterial vaginosis: a case-control study. Iran J Obstet Gynecol Infertility.

[12] Krebs-Smith SM, Pannucci TE, Subar AF, Kirkpatrick SI, Lerman JL, Tooze JA (2018). Update of the healthy eating index: HEI-2015. J Acad Nutr Diet.

[13] Rahmani J, Varkaneh HK, Ryan PM, Zarezadeh M, Rashvand S, Clark C (2019). Healthy Eating Index-2015 as a predictor of ulcerative colitis risk in a case–control cohort. J Dig Dis.

[14] Thoma ME, Klebanoff MA, Rovner AJ, Nansel TR, Neggers Y, Andrews WW (2011). Bacterial vaginosis is associated with variation in dietary indices. J Nutr.

[15] Tachedjian G, Aldunate M, Bradshaw CS, Cone RA (2017). The role of lactic acid production by probiotic Lactobacillus species in vaginal health. Res Microbiol.

[16] Osuna-Padilla IA, Leal-Escobar G, Garza-García CA, Rodríguez-Castellanos FE (2019). Dietary Acid Load: mechanisms and evidence of its health repercussions. Nefrologia (Engl Ed).

[17] Delaney ML, Onderdonk AB, Microbiology, Group PS (2001). Nugent score related to vaginal culture in pregnant women. Obstet Gynecol.

[18] Nugent RP, Krohn MA, Hillier SL (1991). Reliability of diagnosing bacterial vaginosis is improved by a standardized method of gram stain interpretation. J Clin Microbiol.

[19] Money D. The laboratory diagnosis of bacterial vaginosis. Can J Infect Dis Med Microbiol. 2005;16. 10.1155/2005/230319 PMC2095014 18159532

[20] Willett W. Nutritional epidemiology. Third edition. ed. Oxford; New York: Oxford University Press; 2013. ix, 529 pages p.

[21] Mirmiran P, Esfahani FH, Mehrabi Y, Hedayati M, Azizi F (2010). Reliability and relative validity of an FFQ for nutrients in the Tehran lipid and glucose study. Public Health Nutr.

[22] Esmaillzadeh A, Azadbakht L (2008). Major dietary patterns in relation to general obesity and central adiposity among Iranian women. J Nutr.

[23] Mirmiran P, Djazayery A, Hosseini esfahani F, Mehrabi Y, Azizi F (2008). Change in food patterns of Tehrani adults and its association with changes in their body weight and body mass index in District 13 of Tehran: Tehran Lipid and Glucose Study. Iran J Nutr Sci Food Technol.

[24] Kennedy E. Putting the pyramid into action: the Healthy Eating Index and Food Quality Score. Asia Pac J Clin Nutr. 2008;17. 18296305

[25] Remer T, Dimitriou T, Manz F (2003). Dietary potential renal acid load and renal net acid excretion in healthy, free-living children and adolescents. Am J Clin Nutr.

[26] Frassetto LA, Todd KM, Morris RC, Sebastian A (1998). Estimation of net endogenous noncarbonic acid production in humans from diet potassium and protein contents. Am J Clin Nutr.

[27] Shivakoti R, Tuddenham S, Caulfield LE, Murphy C, Robinson C, Ravel J, et al. Dietary macronutrient intake and molecular-bacterial vaginosis: Role of fiber. Clin Nutr. 2020. 10.1016/j.clnu.2020.01.011 PMC7387193 32033845

[28] Collins SL, McMillan A, Seney S, van der Veer C, Kort R, Sumarah MW (2018). Promising prebiotic candidate established by evaluation of lactitol, lactulose, raffinose, and oligofructose for maintenance of a lactobacillus-dominated vaginal microbiota. Appl Environ Microbiol.

[29] Serafini M, Peluso I, Raguzzini A (2010). Flavonoids as anti-inflammatory agents. Proc Nutr Soc.

[30] Kalia N, Singh J, Kaur M (2019). Immunopathology of Recurrent Vulvovaginal Infections: New Aspects and Research Directions. Front Immunol.

[31] Jovanovic-Malinovska R, Kuzmanova S, Winkelhausen E (2014). Oligosaccharide profile in fruits and vegetables as sources of prebiotics and functional foods. Int J Food Prop.

[32] Jeffery IB, O’Toole PW (2013). Diet-microbiota interactions and their implications for healthy living. Nutrients.

[33] Claesson MJ, Jeffery IB, Conde S, Power SE, O’connor EM, Cusack S (2012). Gut microbiota composition correlates with diet and health in the elderly. Nature.

[34] Wong JM, De Souza R, Kendall CW, Emam A, Jenkins DJ (2006). Colonic health: fermentation and short chain fatty acids. J Clin Gastroenterol.

[35] Daïen CI, Pinget GV, Tan JK, Macia L (2017). Detrimental impact of microbiota-accessible carbohydrate-deprived diet on gut and immune homeostasis: an overview. Front Immunol.

[36] Antonio MA, Rabe LK, Hillier SL (2005). Colonization of the rectum by Lactobacillus species and decreased risk of bacterial vaginosis. J Infect Dis.

[37] Sobel JD (2007). Vulvovaginal candidosis. Lancet.

[38] Farhangnia P, Akbarpour M. Immunological Tolerance. 2021.

[39] Mahdavi-Roshan M, Salari A, Kheirkhah J, Ghorbani Z (2022). The Effects of Probiotics on Inflammation, Endothelial Dysfunction, and Atherosclerosis Progression: A Mechanistic Overview. Heart Lung and Circulation.

[40] Neggers YH, Nansel TR, Andrews WW, Schwebke JR, Yu KF, Goldenberg RL (2007). Dietary intake of selected nutrients affects bacterial vaginosis in women. J Nutr.

[41] Delchier N, Herbig AL, Rychlik M, Renard CM (2016). Folates in fruits and vegetables: contents, processing, and stability. Compr Rev Food Sci Food Saf.

[42] García-Closas R, Berenguer A, Tormo MJ, Sánchez MJ, Quiros JR, Navarro C (2004). Dietary sources of vitamin C, vitamin E and specific carotenoids in Spain. Br J Nutr.

[43] Meydani SN, Leka LS, Fine BC, Dallal GE, Keusch GT, Singh MF (2004). Vitamin E and respiratory tract infections in elderly nursing home residents: a randomized controlled trial. JAMA.

[44] Bunout D, Barrera G, Hirsch S, Gattas V, de la Maza MP, Haschke F (2004). Effects of a nutritional supplement on the immune response and cytokine production in free-living Chilean elderly. JPEN J Parenter Enteral Nutr.

[45] Nathens AB, Neff MJ, Jurkovich GJ, Klotz P, Farver K, Ruzinski JT (2002). Randomized, prospective trial of antioxidant supplementation in critically ill surgical patients. Ann Surg.

[46] Al-Ghazzewi F, Tester R (2016). Biotherapeutic agents and vaginal health. J Appl Microbiol.

[47] Parsapure R, Rahimiforushani A, Majlessi F, Montazeri A, Sadeghi R, Garmarudi G. Impact of health-promoting educational intervention on lifestyle (nutrition behaviors, physical activity and mental health) related to vaginal health among reproductive-aged women with vaginitis. Iran Red Crescent Med J. 2016;18(10). 10.5812/ircmj.37698 PMC5292014 28184325

[48] Lau C, Færch K, Glümer C, Tetens I, Pedersen O, Carstensen B (2005). Dietary glycemic index, glycemic load, fiber, simple sugars, and insulin resistance: the Inter99 study. Diabetes Care.

[49] Verstraelen H, Delanghe J, Roelens K, Blot S, Claeys G, Temmerman M (2005). Subclinical iron deficiency is a strong predictor of bacterial vaginosis in early pregnancy. BMC Infect Dis.

[50] Jackson J, Williams R, McEvoy M, MacDonald-Wicks L, Patterson A (2016). Is Higher Consumption of Animal Flesh Foods Associated with Better Iron Status among Adults in Developed Countries?. Syst Rev Nutrients.

[51] Bendich A (2001). Micronutrients in women’s health and immune function. Nutrition.

[52] Bhaskaram P (2001). Immunobiology of mild micronutrient deficiencies. Br J Nutr.

[53] Kumar P, Chatli M, Mehta N, Singh P, Malav O, Verma AK (2017). Meat analogues: Health promising sustainable meat substitutes. Crit Rev Food Sci Nutr.

[54] Noormohammadi M, Eslamian G, Kazemi SN, et al. Association between dietary patterns and bacterial vaginosis: a case–control study. Sci Rep. 2022:12:12199. 10.1038/s41598-022-16505-8. 10.1038/s41598-022-16505-8 PMC9288476 35842517

[55] Singh RK, Chang HW, Yan DI, Lee KM, Ucmak D, Wong K (2017). Influence of diet on the gut microbiome and implications for human health. J Transl Med.

[56] Kelley DS (2001). Modulation of human immune and inflammatory responses by dietary fatty acids. Nutrition.

[57] MIURA S, TSUZUKI Y, HOKARI R, ISHII H (1998). Modulation of intestinal immune system by dietary fat intake: relevance to Crohn’s disease. J Gastroenterol Hepatol.

[58] Meksawan K, Venkatraman JT, Awad AB, Pendergast DR (2004). Effect of dietary fat intake and exercise on inflammatory mediators of the immune system in sedentary men and women. J Am Coll Nutr.

[59] Kawahito S, Kitahata H, Oshita S (2009). Problems associated with glucose toxicity: role of hyperglycemia-induced oxidative stress. World J Gastroenterol.

[60] Mousavi M, Jahromi SR, Togha M (2021). The Association Between Dietary Acid Load and Odds of Migraine: A Case–Control Survey. Neurol Ther.

[61] Sanz Juana Maria. et al. Dietary Acid Load but Not Mediterranean Diet Adherence Score Is Associated With Metabolic and Cardiovascular Health State: A Population Observational Study From Northern Italy. Front nutr. 2022, 617. 10.3389/fnut.2022.828587 PMC9087734 35558749

[62] Lee KW, Shin D (2020). Positive association between dietary acid load and future insulin resistance risk: findings from the Korean Genome and Epidemiology Study. Nutr J.

[63] Rezazadegan M, Mirzaei S, Asadi A (2022). Association between dietary acid load and metabolic health status in overweight and obese adolescents. Sci Rep.

[64] Abshirini M, Bagheri F, Mahaki B (2019). The dietary acid load is higher in subjects with prediabetes who are at greater risk of diabetes: a case–control study. Diabetol Metab Syndr.

[65] Thomas SV, Suresh K, Suresh G (2013). Design and data analysis case-controlled study in clinical research. Ann Indian Acad Neurol.

[66] Althubaiti A (2016). Information bias in health research: definition, pitfalls, and adjustment methods. J Multidiscip Healthc.

